# News and Miscellany

**Published:** 1904-10

**Authors:** 


### News and Miscellany.

TYPOGRAPHICAL ERROR in editorial last month. A dram mark was used instead of an ounce mark, in formula for bromidia. There were others.

---

DOCTOR, show your wife the advertisement of A. F. Hardie, Austin, Texas, in front form in this issue. It will interest you and her and all the children. Splendid stock, latest things; prices right. Our Mascot trades there; so do I.

---

DOCTORS' OFFICES should present a neat and clean appearance. A new floor covering—an Oriental art square—is the dinktum. See advertisement of the Oriental Importing Co., and send for illustrated price list free; a postal card will fetch it. Price—just like finding it in the road. We know; we have two of them. They are beauties.

---

THE SOUTH TEXAS SANITARY ASSOCIATION met at Houston September 19th ult. This association is composed of mayors, health officers, members of city councils, county judges, commissioners, physicians, and, "in general, every white citizen in South Texas interested in sanitation, is eligible to membership." Dr. Frank R. Ross, Houston, is secretary, and Mayor W. S. Gardien, of Gonzales, is president. No report received at time of going to press.

---

### "Out of the Mouths of Babes."

When the May and June baby had got well acquainted, they exchanged confidences.

"My milk comes from a certified cow," said the May baby.

"So does mine," said the June baby.

"It is milked by a man in a white suit, with sterilized hands, through absorbent cotton, and kept at a temperature of forty-five degrees."

"So is mine."

"It is brought to me in a prophylactic wagon drawn by a modified horse."

"So is mine."

"Then how in the thunder do you manage to be so fat and well?"

The June baby winked slyly.

"I chew old paper and the corners of the rugs and anything I can find that is dirty, and in that way I manage to maintain the bacterial balance which is essential to health," he said, chuckling.

The May baby laughed long and loud.

"So do I," said he.

The mamas heard the goo-goo-ing, but they assigned it to only the usual fantastic significance. It was just as well.—*Exchange.*

---

### The Captain and His Craft.

I'm a sailor bold, if there ever was one!
In the nautical line, I'm a son of a gun!
I haven't a crew and I haven't a boat,
And really don't think I was ever afloat!
But still if you'll listen a moment to me,
You'll see that I'm speaking a veritee!
For I am the crew,
And the captain, too,
Of an elegant Floating Kidney!

On days when the yachtsman boards his yacht
And the wind is a-blowing eleven knot,

And I feel the need of a little sport,
I push the kidney away from its port;
Oh, it is a pleasure and it's a pride,
As she lightly lists to the starboard side
And readily goes,
Known only to those
Who are blessed with a Floating Kidney.

I wear in the summer a yachting cap,
And look the part of a nautical chap!
And naught in the world can please me more
Than calling me Cap'n or Commodore!
As little of life and its ills I reck
As an Admiral walking on his quarterdeck!
There's nothing, I hold,
Like the captain bold
Of an elegant Floating Kidney!
—*N. O. Times-Democrat, July 28, 1904.*

---

ANOTHER VERSE BY THE BIG SUNFLOWER.

*For Texas Medical Journal.*

He foolishly thought he would always remain
Like a Commodore, autocrat, the same,
But one day happening to step ashore,
Down upon him a nautical surgeon bore.
"This sailor has gold I need a stake;
Out of his back his floater I'll take."
The sailor has gone
To his long happy home
Without his floating kidney.

---

THE FIFTH DISTRICT MEDICAL SOCIETY held its second semi-annual meeting at San Antonio, August 25, 1904. There was a large attendance and much enthusiasm prevailed. The meeting closed with a "smoker," whereat there was a feast of wit and a flow of Anheuser; toasts were snappy, responses eloquent, and much enjoyed. I reproduce the address of the President, Dr. Malone Duggan, of Eagle Pass. It is eminently sensible.

Dr. Duggan said, in part:

"The American Medical Association has been striving to secure the passage of a bill that provides for a standard of purity of food and drugs. This cause should receive our cordial support. Every physician should feel in duty bound to promote, in every way he can, the passage of this bill. This object can never be attained until the medical profession, as a whole, exerts the necessary influence. It is our right to demand the highest standard of purity in both foods and drugs.

[There is a pure-food law on our statutes, passed in 1885, but it is and ever has been a dead letter for lack of an appropriation to make it effective.—DANIEL.]

"Nothing can be too good for the afflicted who rely upon us for relief. We are working at a great disadvantage when we do not know the character of our drugs, and that we are imposed upon every day is common knowledge to us all. I think a committee from this society who would take the matter up conscientiously and put themselves in line with the work of the Association could accomplish a great deal.

"First having adjusted our own defects and having plucked out the beam that is in our own eyes, we can with propriety proceed to cast out the mote that is in our brother's—the public—eye. The profession owes a duty to the public which has not been discharged as it ought to have been. Perhaps this is due more to the lack of concert of action than to any other cause.

"I first refer to the 'social evil.' No one doubts its peril and the necessity of prompt action. Without discussing the question here, I would recommend that a committee be entrusted to thoroughly investigate this question and to present at our next meeting a clear statement of the case, with suggestions of some plan of action. The same committee could also consider the question of rape and its remedy.

"Another public benefit is the regulation of the practice of medicine. One cause of our failure in the past to secure proper legislation has been the fallacy that the public thought that we were asking legislation for our own interest. Now, let us convince them that this is not so, but that we are only volunteering our services, as guardians of the public health, to secure protection for them. In this matter I believe that each county society, individually, can do more than could a general committee, by exerting their influence among their legislative representatives.

"Quarantine has always been regarded as a most important public necessity, and in considering it here I would ask that if in view of the advances made in the etiology of contagious diseases are the laws regulating quarantine just and equitable? Here we are again the public's guardian, and we must answer for the people. Let a committee sift this question and put the profession right before the public in the report at our next meeting.

"And, lastly, I would regulate what I believe to be a greater peril to the public than the unqualified doctor; a greater peril than the social evil and a greater peril than the danger from smallpox and yellow fever. I refer to the patent medicine craze and the practices of drugless doctors. Under drugless doctors I would include every irregular practice, from Christian Science down through the list.

"Let us have an enthusiastic committee on this subject, who will secure data and present at our next meeting a ringing report on this our most menacing public peril."

The following committees were appointed:

*On the Use by the Profession of Proprietary and Synthetic Preparations*—Drs. J. V. Spring and W. M. Wolf, of San Antonio; J. T. FitzSimon, of Castroville.

*On the Question of Pure Food and Pure Drugs*—Drs. Jas. Hall Bell and J. Braunagel, of San Antonio, and E. G. Palmer, of Kerrville.

*On the "Social Evil"*—Drs. Amos Graves, Jr., and G. H. Moody, of San Antonio, and A. Garwood, of New Braunfels.

*On the Use of Patent Medicines by the Laity and the Practice of Drugless Doctors*—Drs. G. G. Watts and B. F. Kingsley, of San Antonio, and W. M. Clark, of Floresville.

The next meeting will be held in April, 1905.

